# Interaction Between Commensal Bacteria, Immune Response and the Intestinal Barrier in Inflammatory Bowel Disease

**DOI:** 10.3389/fimmu.2021.761981

**Published:** 2021-11-11

**Authors:** Yongyan Chen, Wenwen Cui, Xiao Li, Huan Yang

**Affiliations:** Xuzhou Key Laboratory of Laboratory Diagnostics, School of Medical Technology, Xuzhou Medical University, Xuzhou, China

**Keywords:** commensal bacteria, immune response, intestinal barrier, IBD, SCFAs

## Abstract

In inflammatory bowel disease (IBD), intestinal mucosa cell and intestinal epithelial cell are severely damaged, and then their susceptibility to bacteria increases, so many commensal bacteria become pathogenic. The pathogenic commensal bacteria can stimulate a series of compensatory immune responses in the intestine. However, the immune response prevents the intestinal tract from restoring homeostasis, which in turn produces an indispensable inflammatory response. On the contrary, in IBD, the fierce inflammatory response contributes to the development of IBD. However, the effect of commensal bacteria on inflammation in IBD has not been clearly studied. Therefore, we further summarize the changes brought about by the changes of commensal bacteria to the inflammation of the intestines and their mutual influence. This article reviews the protective mechanism of commensal bacteria in healthy people and the mechanism of commensal bacteria and immune response to the destruction of the intestinal barrier when IBD occurs. The treatment and prevention of IBD are also briefly summarized.

## Introduction

Inflammatory bowel disease (IBD) is chronic and recurrent and it mainly affects the ileum, rectum, and colon. It includes ulcerative colitis (UC) and Crohn’s disease (CD). The global burden of IBD remains a persistent health problem. The prevalence of IBD in Europe, North America, and other Western countries exceeds 0.3% and is increasing in many newly industrialized countries (1, 2).

The affected bowel in UC and CD are quite different. UC is limited to the colon, it mainly invades the lamina propria and crypts (3, 4). CD can cause transmural enteritis, it can affect any part of the stomach and intestine, especially the terminal ileum and colon (5). The damage caused by IBD can be attributed to the destruction of the intestinal barrier (3, 6). The etiology of IBD is not yet fully understood, and it has been proven to be related to complex factors such as genetics, environment, intestinal microbes, and immunity. Among them, intestinal commensal bacteria play a pivotal role, including bacteria and fungi (3, 7).

Intestinal commensal bacteria do not play an independent role in host health. Under normal circumstances, commensal bacteria and the intestinal immunity of the host are in a balanced state, and they resist the invasion of pathogenic microorganisms and maintain homeostasis together (8). The human intestinal barrier is the first line of defense for the invasion of pathogenic microorganisms (9). It is associated with many diseases, such as IBD, acute pancreatitis, colon cancer (10–12). The direct communication between the intestinal symbiotic flora and intestinal immunity controls the development of the disease. Once the balance is broken, enteritis will be induced in the form of positive feedback (13).

The commensal bacteria in IBD always have non-negligible changes, including their quantity or products, and the coordination of the immune response with the intestinal tract exposes the intestinal barrier to danger (14). It causes the activation of immune cells and an overload of cytokines, and the activation of a series of receptors and proteins ultimately promotes the occurrence and development of IBD (15). In addition, oral commensal bacteria also contribute to the development of IBD. They will be highly colonized in the unhealthy and fragile intestines after swallowing. This has been verified. The pathogenic bacteria in these conditions will further threaten the intestinal tract (16). Many risk factors for IBD, such as diet, smoking, and antibiotics (3), can change the state of normal intestinal commensal bacteria and immunity and increase the risk of IBD.

In this review, we focus on discussing the lesions of the colonic mucosa of IBD, which are both present in CD and UC (17). We describe the barrier protection of commensal bacteria and intestinal immunity in a healthy state and focus on the changes in commensal bacteria in IBD and the damage of commensal bacteria-dependent immune responses to the intestinal barrier. Each of these mechanisms is closely linked, with feedback mechanisms to promote the final outcome, IBD. We then discuss the treatments and prevention that rely on this series of mechanisms.

## Commensal Bacteria Maintain the Homeostasis of the Intestinal Mucosal Barrier

The intestinal barrier is mainly composed of mucus and epithelial cells. Commensal bacteria and the immune system in the intestine work together to regulate the homeostasis of the intestinal barrier. The gastrointestinal tract provides 150-200 m^2^ of surface area for microorganisms (18), and about 10^13^ bacteria are colonized, which is comparable to the number of human cells (19). Because of pH and other factors, a small number of microorganisms are colonized above the upper end of the small intestine. The most abundant commensal bacteria are found in the colon, accounting for about 70% of all bacteria in the human body. Most are *Bacteroidetes*, *Firmicutes*, *Actinobacteria*, *Proteobacteria*, and *Verrucomicrobia* (20, 21). There are large differences in individual flora, which are closely related to dietary and living habits (22).

### Mucosal Barrier Isolates Commensal Bacteria and Epithelial Tissue

**Figure 1:**
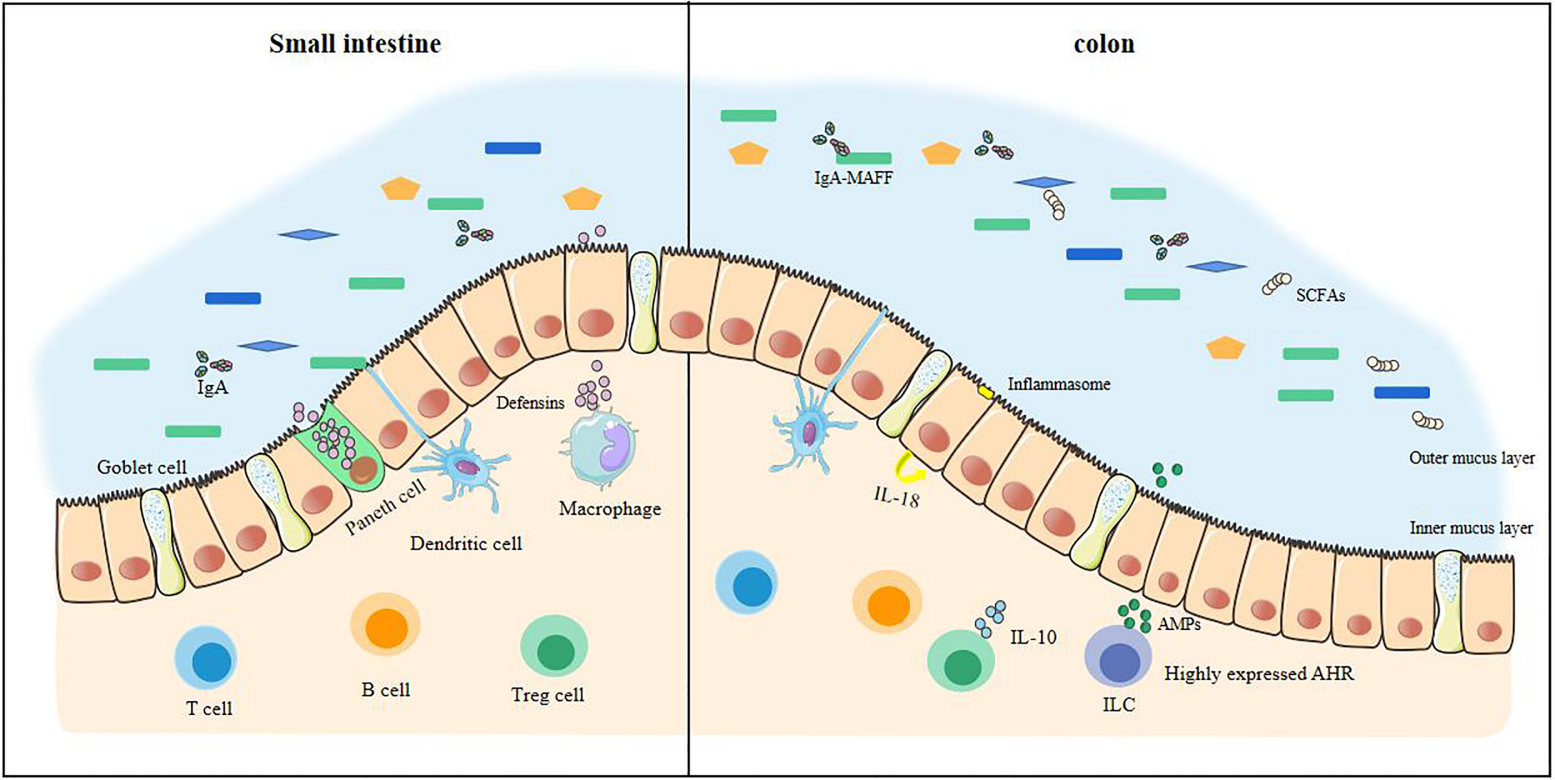
The stable intestinal barrier of a healthy small intestine and colon. The homeostasis of the intestinal tract of a healthy body depends on the regulation of many factors, including commensal bacteria, short-chain fatty acids, and the immune system. In health, commensal bacteria are diverse. The antibacterial substances produced in the small intestine are sufficient to resist pathogenic bacteria, making it difficult to cross the intestinal epithelial cells. There is also an internal mucus layer in the colon that cannot be crossed by these bacteria. The production of short-chain fatty acids keeps the colon in a hypoxic state and maintains tight epithelial junctions, increasing anti-inflammatory effects. At this time, the immune system has a large anti-inflammatory effect, which is enough to prevent the inflammatory load caused by the invasion of pathogenic bacteria.

The intestinal barrier is in constant dynamic renewal (**Figure 1**). Stem cells continue to divide and proliferate, replenishing the epithelial cells shedding into the intestinal lumen and becoming epithelial cells (23). The secretory activity of goblet cells (secretion of mucin, especially MUC2) gives intestinal epithelial cells an extra protective gel mucus layer (24). Commensal bacteria can pass through the mucus in the small intestine (25). When a small amount of commensal bacteria pass through the mucus, they are presented to T cells and B cells by dendritic cells in the intestine and induce B cells to produce IgA, which targets intestinal bacteria. At this time, the macrophages in the lamina propria are activated, and the macrophages exert their phagocytosis and secretion functions. With the production of antibacterial substances such as defensins, the commensal bacteria that are about to touch the epithelial cells can be completely eliminated (26, 27). The defensins secreted by the Paneth cells in the small intestine enhance this effect (28). The mucus in the colon can directly prevent the contact of commensal bacteria, and the outer mucus layer provides a living environment for the commensal bacteria (24, 25), and IgA also exists in a large amount in the outer mucus layer. The IgA of glycosylation, however, no longer resists commensal bacteria. The IgA-MAFF (Mucus-Associated Functional Factor) system can promote the growth of *Bacteroidetes*, induce the proliferation of *Clostridium*, promote the renewal of epithelial cells, and deal with the damage in time, stabilizing the intestinal barrier (29).

### Short-Chain Fatty Acids Are Positive for Stabilizing the Intestines

Short-chain fatty acids (SCFAs) play a pivotal role in maintaining intestinal homeostasis, especially butyrate, the main source of energy for colon cells (30). SCFAs are produced when the commensal bacteria metabolize dietary fiber. *Bacteroidetes* and *Firmicutes* are the most abundant bacteria in the intestine. The *Bacteroidetes* mainly produce acetic acid and propionic acid, while *Firmicutes* mainly produce butyrate in the human intestine (31, 32). Butyrate can maintain the proliferation of small intestinal epithelial cells (33). As butyrate is produced by anaerobic bacteria, butyrate stabilizes hypoxia inducible factor (HIF, which is able to coordinate barrier protection) during the process of being absorbed and metabolized in an hypoxic environment (34, 35) and can induce innate lymphocyte (ILCs) to produce antimicrobial peptides (AMPs) to regulate commensal bacteria (36). The β-oxidation of butyrate can maintain the low-oxygen environment in the intestine so that butyrate in a healthy intestine can be continuously produced (37). SCFAs activate and release IL-18 through inflammasomes to maintain the integrity of the intestinal epithelium (38). In addition, SCFAs also have anti-inflammatory effects (39). They can increase the IgA in the intestine, maintain the development of B cells, and play an important role in promoting the differentiation and expansion of Treg cells (38, 40).

Therefore, commensal bacteria help the intestines resist pathogens at any time, and its function depends on a good ratio of commensal bacteria.

### The Main Immune Factors in a Healthy Intestine That Inhibit Inflammation

In a healthy gut, immunity is the main factor that maintains homeostasis. The proliferation and differentiation of T cells are inhibited, but Treg cells are active. IL-10 may be produced by most immune cells, especially Treg cells. IL-10 directly inhibit the production of IL-12 and IL-23, which is equivalent to limiting the differentiation of Th1 cells and the response of pathogenic Th17 cells (41). IL-10 deficiency mainly induce Th17 cells, and then Th17 could promote colitis (42). The activation of the aryl hydrocarbon receptor (AHR) is another mechanism of intestinal homeostasis. Once activated, AHR releases IL-22, induces IL-10R expression, and strengthens the tight junctions of the intestinal epithelium to maintain the integrity of the intestine (43). AHR is highly expressed in ILC2 and can inhibit the expression of IL-33 receptor ST2 [this receptor is highly expressed in IBD (44)] and the production of some pro-inflammatory factors, such as IL-13 (45). Overall, the homeostasis of the intestine needs to be maintained by inhibiting inflammatory factors to prevent excessive inflammation. When the intestinal commensal bacteria change, the “balance” of immunity will be broken and tilted toward proinflammatory effects, and the body will be in a state of inflammatory stress.

## Interaction Between Commensal Bacteria and the Intestinal Barrier in IBD

### Commensal Bacteria Degrade More Mucus

**Figure 2:**
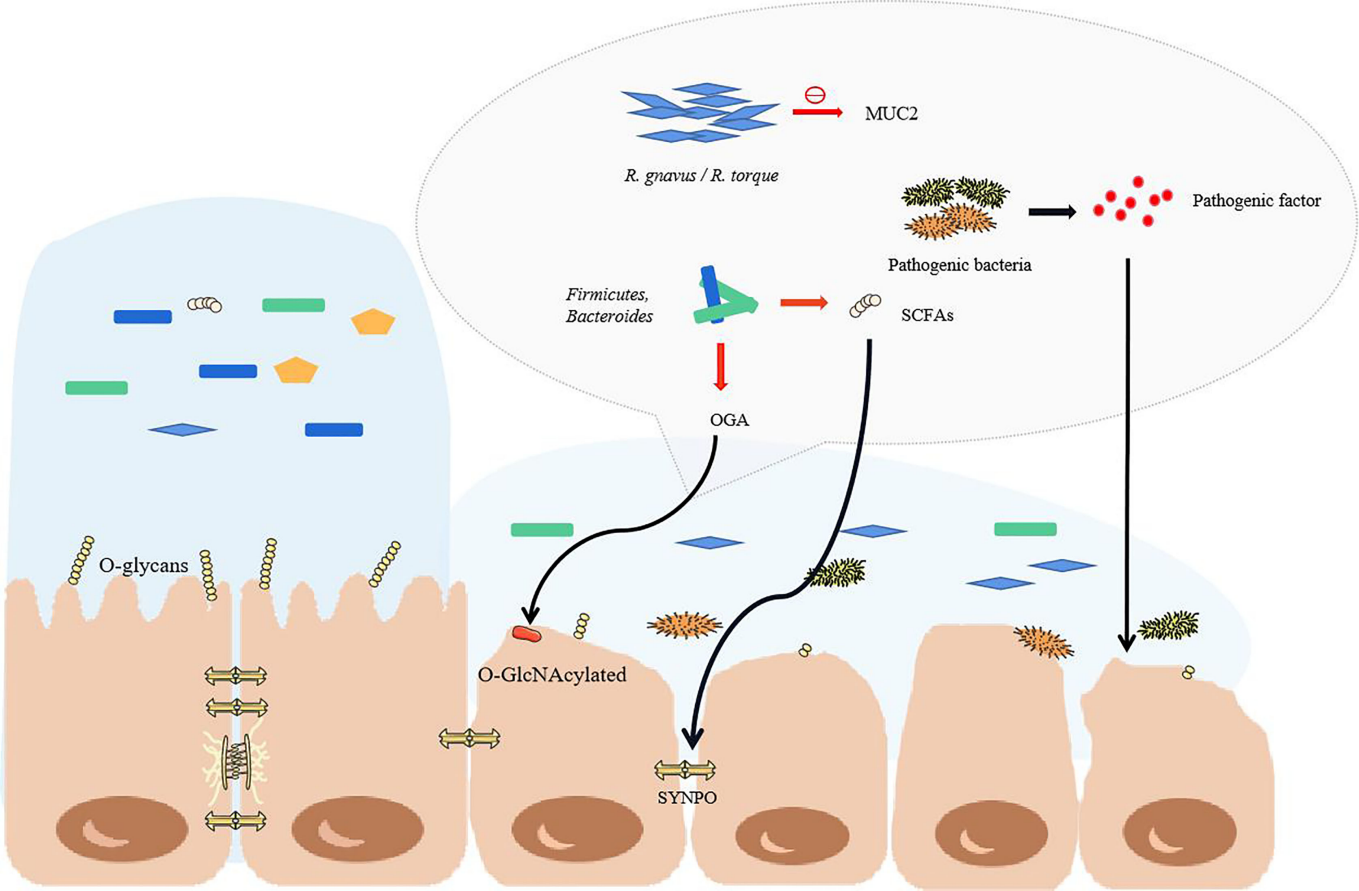
The structural changes of commensal bacteria during IBD lead to the destruction of the intestinal barrier. When the structure of the commensal bacteria changes, the mucus in the colon becomes thinner, mainly due to the increase in the proportion of some major mucus-degrading bacteria. The O-glycans on the intestinal epithelial cells would normally stabilize the mucus, but they are reduced in IBD. At this time, goblet cells cannot differentiate normally, so the mucus becomes thinner, and pathogenic bacteria enter the inner mucus layer and contact the epithelium. As the proportion of pathogenic bacteria greatly increases, the pathogenic factors produced directly attack the intestinal cells. With the reduction of short-chain fatty acid-producing bacteria, the tight junctions of the intestine cannot be maintained. The red arrow highlights the reduction.

During IBD, the microbial genes detected in feces were reduced by about 25%, and the abundance and diversity of commensal bacteria decreased overall (46, 47). Patients with IBD have more harmful bacteria and fewer beneficial bacteria. In fact, under the protection of the mucus barrier, the intestinal tract can maintain a steady state even though the bacteria in the intestinal tract undergo minor changes (48). However, the total abundance of mucus-degrading bacteria in IBD patients is increased significantly (49). The mucus-degrading bacteria in healthy humans degrade the skeletal structure of mucus-mucin (MUC2) to release the product for use by other bacteria (50), but the sharp increase in mucus-degrading bacteria, such as *Ruminococcus gnavus* (*R. gnavus*) and *Ruminococcus torques* (*R. torque*), will dissolve more mucus and increase the exposure of epithelial cells (49). In addition, IBD is usually accompanied by truncated O-glycans in intestinal epithelial cells, which may be related to glycosidase produced by microorganisms and oxidative stress caused by inflammation (51). The truncated intestinal sugar chain makes the mucosa thinner, and the SCFAs are reduced in IBD and cannot regulate mucin glycosylation normally (52). This makes mucin lose its stability. The decreased *Bacteroides* and *Faecalibacterium prausnitzii* also failed to promote the differentiation of goblet cells, and they could not regulate the glycosylation of the mucosa (**Figure 2**) (53–55).

### The Structure of Commensal Bacteria Is Changed in IBD

With the increase of *R. gnavus* and *R. torque*, more mucosa is broken down, and the inflammation of IBD makes the intestinal mucosa become thinner. The change in oxygen gradient is conducive to the survival of intestinal facultative aerobes and reduces the proportion of anaerobic bacteria. This should be the main reason for the changes in the structure of intestinal commensal bacteria (**Figure 2**) (56, 57). Therefore, the thin intestinal mucus cannot stop the invasion of the increased pathogenic bacteria. *Proteobacteria*, *Escherichia coli*, *Fusobacteria*, *Klebsiella pneumoniae*, *Clostridium difficile* and other pathogenic commensal bacteria in the intestine of patients with IBD are increased significantly (58–61). This is an extremely serious phenomenon for intestinal homeostasis. Among them, *Klebsiella pneumoniae* is most likely swallowed through the mouth to the intestines and colonized, which may be before or after IBD. In fact, oral commensal bacteria take advantage of IBD to take advantage of it. At this time, the colonization resistance of the intestinal tract of a healthy body no longer exists. Ectopic colonization of conditional pathogens, including *Porphyromonas gingivalis*, *Streptococcus mutans*, *Fusobacterium nucleatum*, *Campylobacter concisus*, and *Klebsiella pneumoniae*, will aggravate the development of IBD (15, 62).

*Proteus* has a low abundance in healthy intestines and are the first reported pathogenic bacteria in gastrointestinal diseases. Because of its adhesion and ability to produce urease, hemolysin, and virulence factors, its pathogenicity is significantly manifested in IBD with the increase in number (63, 64). Adherent invasive *Escherichia coli* (AIEC) is a well-known pathogen. AIEC penetrates the mucus layer and resists antibacterial proteins, adheres to intestinal epithelial cells to release enterotoxins, and can block the autophagy process of lysosomes (autophagy plays an important role in maintaining the body’s immune homeostasis) (65, 66). For patients with IBD who lack *PTPN2* (an autoimmune susceptibility gene) or mutations in *PTPN2*, this is more like a help. Macrophages won’t against AIEC (67). At the same time as the increase in pathogenic bacteria, the proportion of beneficial commensal bacteria such as *Firmicutes*, *Clostridiales*, *Bacteroides*, *Ruminococcaceae, Lactobacillus*, *Bifidobacteria*, *Faecalibacterium prausnitzii* and other butyrate- producing bacteria in the intestine is significantly reduced, which is also an important factor in the development of IBD (53, 54, 68). O-GlcNAcase (OGA) that is enriched in *Bacteroides* and *Firmicutes* can hydrolyze O-GlcNAcylated protein in epithelial cells and immune cells, which can inhibit the development of colitis. However, because of the reduction of bacteria, the level of OGA in IBD decreases, which is also an important mechanism for the continuous development of IBD (69). Indeed, the diversity of intestinal commensal bacteria decreases, especially anaerobic bacteria. In this case, there is no surprise that SCFAs, well-known nutritional and anti-inflammatory substances in the intestine, are drastically reduced, especially butyrate (37, 40). The actin-binding protein synaptopodin (SYNPO) is an important protein that maintains the tight junctions of the intestinal epithelium and is interdependent with butyrate. Therefore, SYNPO in IBD is naturally lost with the decrease of butyrate, which directly affects the connections intestinal epithelium (70).

In recent years, scientists have discovered that fungi also coordinates commensal bacteria, and there is an intricate relationship between them. Although the bacterial changes of UC and CD are similar, fungi are very different. This is mainly related to the ileum due to the unique function of the ileum, it can produce antimicrobial peptides and absorb bile acids. The ileum CD is conducive to the growth of fungi, while the diversity of UC and CD fungi that do not involve the ileum is reduced. This is also inseparable from some commensal bacteria in the intestine. The abundance of *bifidobacteria* and *brucella* in IBD is positively correlated with yeast, but these bacteria are reduced in IBD. In fact, bacteria and fungi are two mechanisms in CD, but they are closely related in UC (71, 72). The common mechanism in IBD is that the ratio of Basidiomycetes/Ascomycetes increases, the ratio of *Candida albicans* increases, and the ratio of *Saccharomyces cerevisiae* decreases, which reduces AIEC-induced colitis (72, 73).

## Interaction Between Commensal Bacteria and Immune Response in IBD

### Innate Immune Response

Dendritic cells form an extensive network under the intestinal epithelium. After a large number of Proteobacteria pass through the mucosal barrier, pathogen-associated molecular patterns (PAMP), lipopolysaccharide and flagellin on the surface of the proteobacteria, are recognized by the toll-like receptors on the surface of dendritic cells (74, 75). The immature dendritic cells produce IL-23, which causes local intestinal inflammation (76). At the same time, macrophages have a phagocytic effect. In addition, the production of cytokines such as IL-1β, IL-6, IL-18, and TNF by dendritic cells and macrophages further aggravates inflammation (77). In addition, IL-10, which inhibits inflammation, is produced by intestinal dendritic cells. *Bifidobacteria* can increase its release, but this is inhibited in IBD by the decrease of these bacteria. Its related *Saccharomyces cerevisiae* also promotes this effect (72, 78). The inflammatory overload caused by macrophages plays a pivotal role in the immune response in IBD, and treatment for it may be able to effectively alleviate the inflammation of IBD (79).

**Figure 3:**
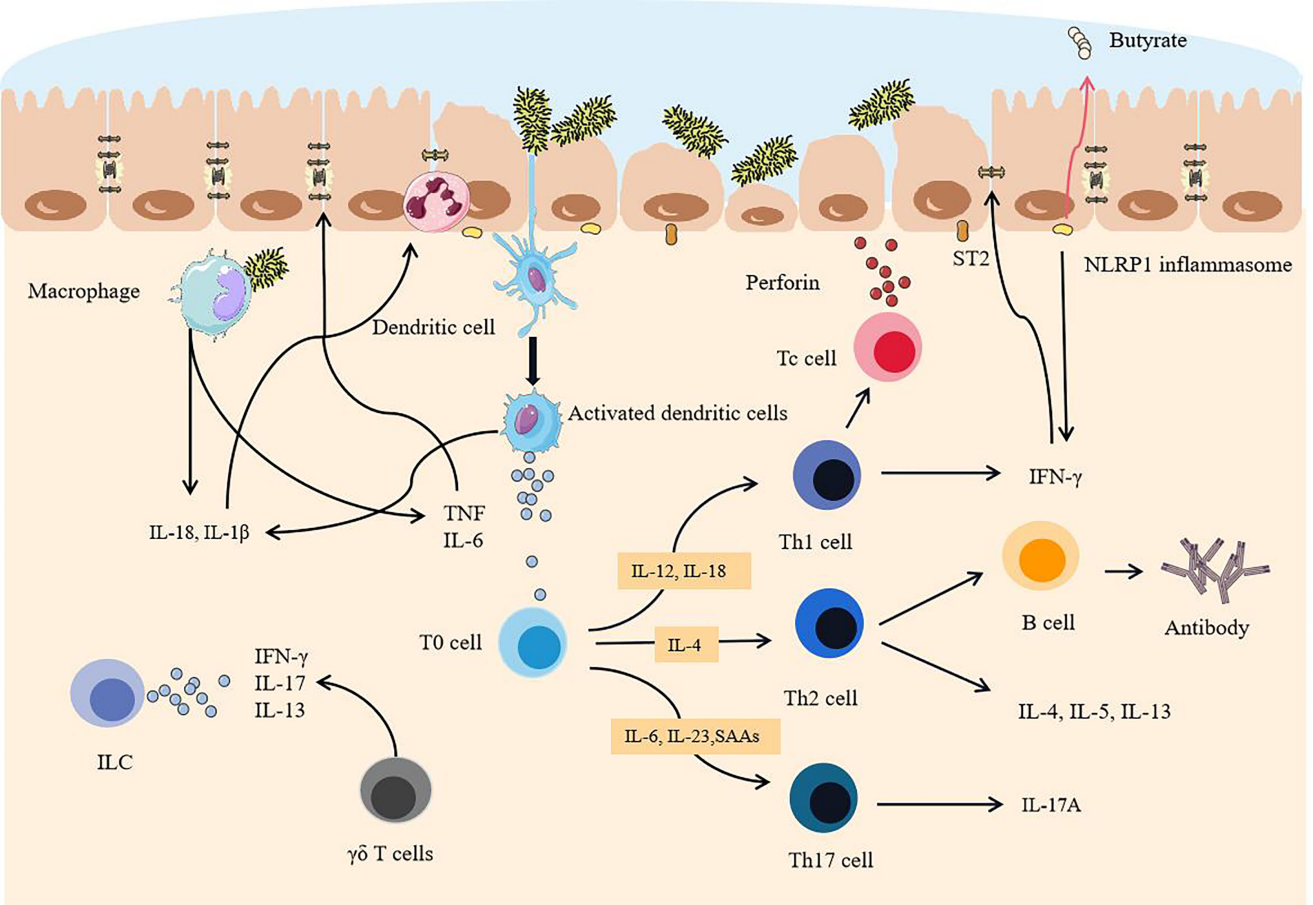
After the intestinal mucosa are degraded, pathogenic commensal bacteria trigger intestinal overload immunity. When the mucosa become thinner, a sharp increase in pathogenic commensal bacteria activates dendritic cells. Because of the destruction of the tight junctions of the intestine, pathogenic commensal bacteria cross the epithelial layer and reach the lamina propria, activating macrophages and NK cells and releasing a large number of cytokines. APC binds antigens to T cells and B cells and differentiates and matures in an environment rich in cytokines. Eventually, a large number of activated immune cells and a large number of proinflammatory factors are dominant, exceeding the body’s ability to inhibit inflammatory effects, epithelial cells are severely attacked, and tight junctions are further destroyed.

Natural killer (NK) cells are activated by cytokines, bind to infected epithelial cells, release toxic particles, and induce apoptosis. IL-1β further promotes the production of IFN-γ, IL-17, and other cytokines by ILCs, and promotes the development of inflammation (80). γδ T cells are another way to produce IL-17 (81), but it can be inhibited by propionate, a metabolite of symbiotic bacteria. However, because of the lack of SCFA-producing bacteria, this effect is also inhibited, which is also one of the reasons for the rampant inflammatory factors. IL-13 can be produced by ILC2 dependent on commensal bacteria (82). A large number of cytokines produced by antigen-presenting cells (APC) not only bring about an overload of inflammation but also provide conditions for specific immune responses (**Figure 3**).

### Adaptive Immune Response

Dendritic cells activate T cells after ingesting antigens, and Th0 cells differentiate into Th1 cells, Th2 cells, and Th17 cells. Th1 cells induce cytotoxic T cells to activate, proliferate, and attack the infected intestinal epithelial cells. Th2 cells activate B cells, proliferate and differentiate into plasma cells, and secrete antibodies to neutralize pathogens. Th17 cells mainly secrete IL-17A to mediate inflammation (83). Moreover, Th1 cells are induced to produce a large amount of IFN-γ by IL-12 and IL-18, which mainly induces CD (84). Th2 cells release IL-4, IL-5, and IL-13 (85), which mainly play a role in UC. Th2 cells can also activate B cells as well and secrete antibodies (mainly IgG) to attack infected epithelial cells, but this response seems to be secondary (84, 86). SCFAs can induce the production of anti-inflammatory Treg cells and the c-MAF transcription factor produced by Treg cells that can maintain the function of Tregs and inhibit the activation of Th17 cells (87). However, because of the reduction of anaerobic bacteria and the lack of anaerobic environment, SCFAs are greatly reduced. Not only that, the colonization of segmented filamentous bacteria in IBD can trigger the secretion of serum amyloid A proteins (SAA1 and SAA2). The increase of these two has been confirmed in IBD, which also promotes the differentiation of pathogenic Th17 cells (88, 89). Conversely, RORγt+Treg in IBD was reduced, which is all due to changes in commensal bacteria (90). For example, *Bacteroides fragilis* is reduced in IBD. It normally acts on immune cells through outer membrane vesicles and relies on the IBD-related gene *ATG16L1*. Because of the lack of this gene and these bacteria, the Treg cell response is defective, which also leads to the inhibition of IL-10 release (91–93). Therefore, once inflammation develops, the axis of Th17 and Treg is destined to tilt, biasing the role of Th17 (94). When oral inflammation such as periodontitis occurs, the pathogenic commensal bacteria expand and at the same time produce Th17 cells and migrate to the intestine (95), which will only aggravate the development of IBD and cause the inflammation of the intestines to persist. Recently, studies have found that tissue-resident memory T cells (TRM cells) play an important role in promoting the development of inflammation in IBD (96). Although the mechanism is not yet clear, it is still very likely to become an effective therapeutic target in the future. In general, as the immune system of the lamina propria is successively activated, the body can no longer regulate inflammatory effect brought by a large number of inflammatory cells and inflammatory factors, which is accompanied by irreversible destruction of the intestinal barrier (97).

### Cytokines Destroy Tight Junctions

A large number of proinflammatory factors produced at this time not only increase the burden of inflammation but also seriously affect the tight junctions of cells. IL-1β can recruit granulocytes to infiltrate the infection foci and directly destroy the connection and tightness of intestinal epithelial cells (98). IL-13 can activate STAT6 in epithelial cells and affect the tight junctions of the intestinal epithelium (82). In addition, TNF-α and IL-1β induce endoplasmic reticulum stress, affect Caco-2 cells (intestinal epithelial cells), and significantly change key proteins from the apical and basolateral membranes, such as E-cadherin. This further destroys the tight junctions of the intestinal epithelium (99). TNF will promote the expression of myosin light chain kinase (MLCK) in the intestinal epithelium, which is also one of the mechanisms by which the permeability of intestinal epithelial tight junctions increases (100). The NLRP1 inflammasome in the intestinal inflammation area of IBD patients increases sharply, which is a known negative factor for butyrate-producing commensal bacteria, and the promoted IFN-γ will promote IBD (101). IFN-γ and IL-13 can induce apoptosis of intestinal epithelial cells and further increase intestinal permeability (102, 103). In addition, after AIEC colonization, AIEC can also induce the expression of IL-33 receptor ST2 in intestinal epithelial cells, thereby enhancing the IL-33 signaling pathway, up-regulating TGF-β, promoting the expression of collagen in fibroblasts, and promoting the development of intestinal fibrosis (44). Once the immune system is activated, the cytokines produced will further attack the tight junctions of intestinal epithelial cells, severely exceeding the self-healing ability of the intestinal barrier.

## Treatment and Prevention

**Figure 4:**
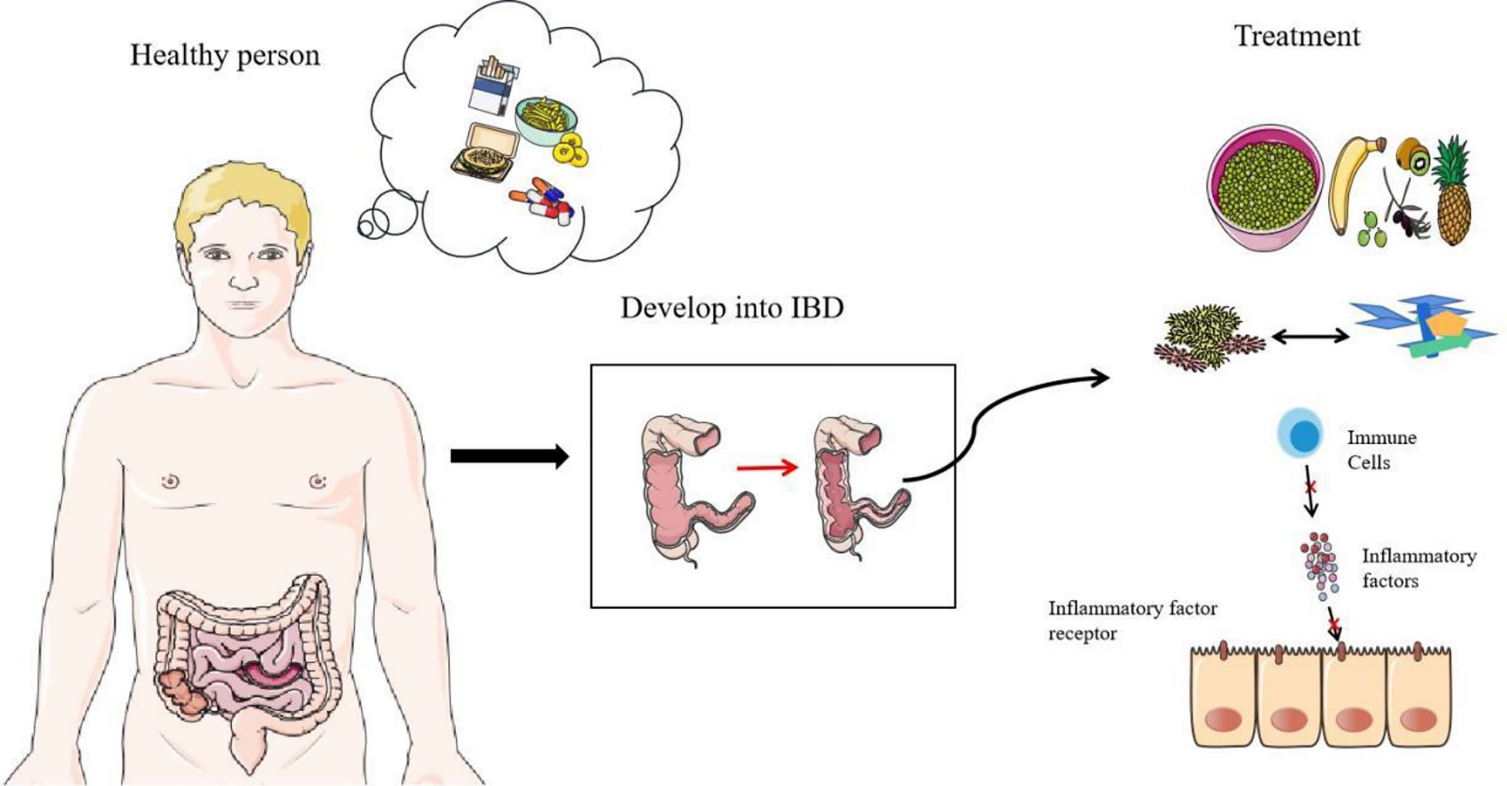
Prevention and treatment of IBD. Diet, smoking, and antibiotics are all predisposing factors for IBD. The first thing they destroy is the normal structure of commensal bacteria. With the research on the mechanism of IBD, there are some therapeutic targets of IBD, including the regulation of diet, the replacement of flora (including FMT and probiotics), and the suppression of immunity (including antagonistic cytokines, antagonistic cytokine receptors and other measures).

In order to improve the balance of commensal bacteria, fecal microbiota transplantation has great potential (104). It seems that the direct replacement of the microbial system in the patient’s body can directly treat IBD, using the healthy microbiota to reestablish a more perfect intestinal barrier. However, because the underlying mechanism of IBD-related core microbiota and pathogenesis is not fully clear, this method still needs further research. Most importantly, this method is not completely safe. Two IBD patients had persistent diarrhea after Fecal microbiota transplantation (FMT) treatment, and enterotoxigenic *C. perfringens* type A was detected in the stool (105). At the end of 2018, a patient was infected with extended-spectrum beta-lactamase-producing *Escherichia coli* after FMT treatment and eventually died (106). In addition, probiotics can also be used as therapeutic agents to treat IBD, but it seems that its efficacy on CD still needs to be explored (107). Scientists are trying to use the metabolites of commensal bacteria as therapeutic targets, such as the use of commensal bacteria that specifically produce butyrate, which has very great therapeutic potential (108). Researchers are also trying to block the overloaded immune response in IBD. Many new drugs reduce the inflammatory response by preventing immune cell migration and communication, such as anti-integrins, anti-MAdCAM-1, and anticytokines. Antibody blocking of the IL-6 signal shows considerable therapeutic effect in the treatment of CD patients (109, 110). ILCs that release a variety of proinflammatory cytokines may also be a new therapeutic target (111). Recently, studies have found that the calcium channel TRPV1 is significantly upregulated in the inflammatory colon of IBD, which may also be a therapeutic target for IBD (112). In addition, proteins that affect intestinal epithelial connections could become therapeutic targets, including NLRP3 inflammasome, STAT6, and MLCK (82, 100, 113). Methods that directly target the mucosa are also being explored, such as stem cell transplantation (114). A high-fiber diet is extremely beneficial for glycosylation of the intestinal mucosa. Strengthening the intestinal barrier seems to be more promising than simply inhibiting the development of inflammation (55). More scientists are also paying attention to dietary therapy and improving the composition of commensal bacteria in the intestine through food intake, which could become an effective treatment plan in the future (**Figure 4**) (115).

The main risk factors for IBD are diet, smoking, and antibiotics (**Figure 4**). Diet is a key factor in the development of IBD. High-calorie food rich in saturated fats, carbohydrates, and animal protein has a negative impact on the composition of intestinal microorganisms (116). A decrease in fiber intake leads to a decrease in the number of commensal bacteria that ferment dietary fiber, and SCFAs are derived from the decomposition of dietary fiber and resistant starch. Diet’s positive effect on anti-inflammatories is well known (117). Regional differences in diet highlight its effect in IBD. The incidence of IBD in many western countries with low-fiber diets is significantly higher than incidence in the eastern countries with high-fiber diets (118, 119). In addition, a high-salt diet will reduce the proportion of Enterobacteriaceae and affect the intestinal ecological balance (120). Smoking and antibiotics are both inducers and promoters of IBD. Studies have shown that they all start by affecting commensal bacteria and disrupting the balance of the intestinal barrier (121–123). Smoking has made a great contribution to increasing *Clostridium*, reducing *Firmicutes*, segmented filamentous bacteria. Smoking has a significant effect on mucin and inflammation genes (124). Besides, antibiotics are susceptible to AIEC in host (125). Overall, the best way to prevent IBD is to form good habits. Quitting smoking and adopting high-fiber diets need to be promoted. A healthy diet may be the best prevention. The correct use of antibiotics also requires our focus. In addition, oral health also requires our attention. Avoid causing or advancing the development of IBD due to tooth decay or periodontitis (62).

## Summary and Outlook

Intestinal mucus separates commensal bacteria from intestinal epithelial cells, which keeps intestinal epithelial cells tightly aligned. The commensal bacteria degrade food in the intestine to supply energy to cells, and the degradation products SCFAs play a major role in immune regulation and reducing inflammation in the intestine. However, when some risk factors destroy the balance of the intestinal tract, the polymorphism and abundance of commensal bacteria reduce, the mucus cannot maintain the normal thickness, and the permeability increases, which allows invasive bacteria to contact intestinal epithelial cells, and induce a series of immune reactions. A large number of immune cells are activated, and the production of immune factors exceeds the limit that the body can maintain. The most prominent effect of IBD is the decrease of beneficial bacteria and the increase of pathogenic bacteria. Understanding the effect of commensal bacteria in the healthy gut and IBD and the dysregulated immune response can lead to developing new therapeutic strategies. In addition, we should pay attention to the risk factors of IBD, promote healthy diet and lifestyle, and establish healthy and strong intestinal barriers and commensal bacteria systems. In the future, scientists should pay more attention to the mutual regulation between commensal bacteria and immune system and explore more effective treatment options. Additional mechanisms of commensal bacteria and the immune system need to be studied.

## Author Contributions

These authors contributed equally to this work. YC wrote the article, WC revised the article, HY provided the theme and made revisions, and XL provided revision opinions. All authors contributed to the article and approved the submitted version.

## Funding

This research was supported by the National Natural Science Foundation of China (82102408), the Xuzhou Science and Technology planning Project (KC20116), Natural Science Research Project of Higher Education Institutions in Jiangsu Province (20KJB310013) and Xuzhou Medical University Excellent Talent Introduction Project (D2019030, RC20552061).

## Conflict of Interest

The authors declare that the research was conducted in the absence of any commercial or financial relationships that could be construed as a potential conflict of interest.

## Publisher’s Note

All claims expressed in this article are solely those of the authors and do not necessarily represent those of their affiliated organizations, or those of the publisher, the editors and the reviewers. Any product that may be evaluated in this article, or claim that may be made by its manufacturer, is not guaranteed or endorsed by the publisher.
